# Comparison of the Impact of Zika and Dengue Virus Infection, and Other Acute Illnesses of Unidentified Origin on Cognitive Functions in a Prospective Cohort in Chiapas Mexico

**DOI:** 10.3389/fneur.2021.631801

**Published:** 2021-03-22

**Authors:** Pablo F. Belaunzarán-Zamudio, Ana M. Ortega-Villa, Alberto J. Mimenza-Alvarado, Paola Del Carmen Guerra-De-Blas, Sara G. Aguilar-Navarro, Jesús Sepúlveda-Delgado, Sally Hunsberger, Raydel Valdés Salgado, José Ramos-Castañeda, Héctor Armando Rincón León, Paul Rodríguez de La Rosa, José Gabriel Nájera Cancino, John Beigel, Sandra Caballero Sosa, Emilia Ruiz Hernández, John H. Powers, Guillermo M. Ruiz-Palacios, Clifford Lane

**Affiliations:** ^1^Departamento de Infectología, Instituto Nacional de Ciencias Médicas y Nutrición Salvador Zubirán, Mexico City, Mexico; ^2^National Institute of Allergy and Infectious Diseases, National Institutes of Health, Bethesda, MD, United States; ^3^Biostatistics Research Branch, Division of Clinical Research, National Institute of Allergy and Infectious Diseases, National Institutes of Health, Bethesda, MD, United States; ^4^Department of Geriatric Medicine, Instituto Nacional de Ciencias Médicas y Nutrición Salvador Zubirán, Mexico City, Mexico; ^5^Geriatrics & Neurology Fellowship, Instituto Nacional de Ciencias Médicas y Nutrición Salvador Zubirán, Mexico City, Mexico; ^6^The Mexican Emerging Infectious Diseases Clinical Research Network (LaRed), Mexico City, Mexico; ^7^Directorate of Research, Hospital Regional de Alta Especialidad Ciudad Salud, Tapachula & Medical Science Research, Hospital General de Zona 1, Instituto Mexicano del Seguro Social, Mexico City, Mexico; ^8^Westat, Rockville, MD, United States; ^9^Departamento de Inmunidad, Instituto Nacional de Salud Pública, Cuernavaca, Mexico; ^10^Instituto Mexicano del Seguro Social, Delegación Estatal, Tapachula, Mexico; ^11^Directorate of Neurology, Hospital Regional de Alta Especialidad Ciudad Salud, Tapachula, Mexico; ^12^Clínica Hospital Dr. Roberto Nettel Flores, Instituto de Seguridad y Servicios Sociales de los Trabajadores del Estado, Tapachula, Mexico; ^13^Departamento de Urgencias, Hospital General de Tapachula, Tapachula, Mexico; ^14^Clinical Research Directorate, Frederick National Laboratory for Cancer Research, Frederick, MD, United States

**Keywords:** humans, mental status and dementia tests, communicable diseases, dengue virus infection, memory, cognition, montreal cognitive assessment, zika virus infection

## Abstract

Zika has been associated with a variety of severe neurologic manifestations including meningitis and encephalitis. We hypothesized that it may also cause mild to subclinical neurocognitive alterations during acute infection or over the long term. In this observational cohort study, we explored whether Zika cause subclinical or mild neurocognitive alterations, estimate its frequency and duration, and compare it to other acute illnesses in a cohort of people with suspected Zika infection, in the region of Tapachula in Chiapas, Mexico during 2016–2018. We enrolled patients who were at least 12 years old with suspected Zika virus infection and followed them up for 6 months. During each visit participants underwent a complete clinical exam, including a screening test for neurocognitive dysfunction (Montreal Cognitive Assessment score). We enrolled 406 patients [37 with Zika, 73 with dengue and 296 with other acute illnesses of unidentified origin (AIUO)]. We observed a mild and transient impact over cognitive functions in patients with Zika, dengue and with other AIUO. The probability of having an abnormal MoCA score (<26 points) was significantly higher in patients with Zika and AIUO than in those with dengue. Patients with Zika and AIUO had lower memory scores than patients with dengue (Zika vs. Dengue: −0.378, 95% CI−0.678 to −0.078; *p* = 0.014: Zika vs. AIUO 0.264, 95% CI 0.059, 0.469; *p* = 0.012). The low memory performance in patients with Zika and AIUO accounts for most of the differences in the overall MoCA score when compared with patients with dengue. Our results show a decrease in cognitive function during acute illness and provides no evidence to support the hypothesis that Zika might cause neurocognitive alterations longer than the period of acute infection or different to other infectious diseases. While effects on memory or perhaps other cognitive functions over the long term are possible, larger studies using more refined tools for neurocognitive functioning assessment are needed to identify these.

**Trial Registration:** NCT02831699.

## Introduction

Zika virus (ZIKV) infection has been associated with severe neurological disease in adults (1). The most frequently identified neurological manifestation is Guillain-Barre syndrome (GBS), which has been estimated to occur in 0.31 to 9.35 per 100,000 people during Zika outbreaks in Latin America and the Caribbean (2). Zika-associated GBS appears to have unique pathophysiological and clinical characteristics, such as the predominance of axonal demyelinating disease and a higher frequency of atypical varieties such as Miller-Fisher syndrome and descending paralysis patterns (3, 4). Along with GBS, ZIKV infection has been associated with other central and peripheral nervous system disorders including meningitis, encephalitis, meningoencephalitis, traverse myelitis, radiculitis, chronic inflammatory demyelinating polyneuropathy, and acute demyelinating encephalomyelitis, optical neuritis, acute hearing loss and other cranial, and peripheral nerve neuropathies (3, 5). ZIKV infect dorsal root ganglion neurons, gastrointestinal tract neurons, and CNS neural progenitors; induce apoptosis and downregulates nucleosome-associated genes, thus decreases cell viability, and enables viral replication (6), which explains neural tube defects, most notably microcephaly, in children of women infected during the first trimester of pregnancy (7).

Although experimental models and human studies have shown that ZIKV has an exquisite affinity for human neural progenitor cells that might explain pre-natal neural damage (8), the mechanisms that induce neuronal damage and explains other neurological manifestations in adults are not clearly understood. Further, severe manifestations in adults and infants have received considerable attention given its conspicuousness and ramifications but it is reasonable to hypothesize that ZIKV infection may cause subclinical or mild disease, which might include neurocognitive impairment during acute infection or over the long term (9–11). In this study, we aimed to explore whether ZIKV infection may cause subclinical or mild neurocognitive alterations, estimate its frequency and duration, and to compare it to other acute illnesses in a cohort of people with suspected Zika infection in Mexico.

## Methods

### Study Design, Study Population and Settings

We analyzed data from the ZIk01 study (https://www.redmexei.mx/) which was a prospective, observational cohort that enrolled patients with probable ZIKV infection and followed them up for 6 months. The study procedures has been described previously (12, 13). Briefly, participants were assessed at enrollment and 3, 7, 28, and 180 days later. During each visit participants underwent a complete clinical exam, including a screening test for neurocognitive dysfunction, a disability assessment, and complete blood count and clinical chemistry. Blood and urine samples for viral nucleic acid identification were also drawn. Participants had their first sample within 7 days after symptoms onset as recommended by the CDC guidance for Zika (14).

Patients were accrued in four clinical care centers in the city of Tapachula in the State of Chiapas, in Southern Mexico from June 2016 to July 2018. We decided to enroll up to 600 participants across three different cohorts (symptomatic people seeking care for symptoms compatible with Zika, patients with Guillain-Barre Syndrome, and asymptomatic household contacts of symptomatic participants) based on convenience and feasibility given the uncertainty of the number of people that would be infected when we planned the study. The protocol specified that separate analyses would be performed for each cohort. This paper analyses data from the cohort of symptomatic participants. In this analysis, we included patients who were at least 12 years old with any two of the following symptoms with onset in the previous 7 days: rash, elevated body temperature (>37.2°C), arthralgia, myalgia, non-purulent conjunctivitis, conjunctival hyperemia, headache, or malaise; not explained by other medical diagnosis, based on a modified version of the Pan American Health Organization case definition (15).

### Study Definitions and Procedures

We classified participants as having confirmed ZIKV, or dengue virus infection if viral RNA was detected in serum or urine samples at enrollment or anytime at 3, 7, or 28 days later. Real-time RT-PCR assays for ZIKV (16), dengue (17), chikungunya (18), and panflavivirus (19) were performed in blood and urine samples from baseline visits and 3,7, and 28 days later. We extracted total nucleic acids from 500 μl of serum and urine using the NucliSENS® easyMAG® system (bioMerieux®, Netherlands) and eluted in 55 μl, according to manufacturer instructions. The amplification of the human RNaseP (RP) gene was carried out for each sample as an internal control to demonstrate the presence of RNA and the validation of the extraction process. The amplification of the NS5 gene was also carried out for the generic detection of Flavivirus as another control of ZIKV and Dengue, and to determine the possible presence of other flaviviruses in the sample. Amplifications were performed in singleplex (each virus detected in a separate reaction) by one-step RT-PCR reaction in 25 μl with SuperScript III Platinum One-Step quantitative RT-PCR System (Invitrogen®, ThermoFisher Scientific®, Waltham, MA, USA) and 5 μl of sample. Cycle sequencing was: retrotranscription at 50°C for 30 min, initial PCR denaturation at 94°C for 2 min followed by 45 cycles of denaturation at 94°C for 15 s and annealing and extension at 60°C for 1 min in the Applied Biosystems 7,500 Fast Real Time PCR System (Applied Biosystems, ThermoFisher Scientific®, Waltham, MA, USA).

Patients with no detectable viral RNA were classified as having an acute illness of unidentified origin (AIUO). We excluded from the analysis 39 participants who had missing data on at least two follow up visits and did not have detectable viral RNA in the available urine or blood samples. We also excluded one participant with confirmed chikungunya virus infection.

We used the Montreal Cognitive Assessment (MoCA) screening tool to assess neurocognitive functions. The MoCA is a one-page test that can be administered in 10 min. The test assesses five domains: (1) memory, (2) visuospatial abilities and executive functions, (3) attention, (4) language, and (5) orientation in time and space (20). Each domain is scored and added for a total score ranging from 0 to 30 points. The Spanish version of the test has been validated in Mexico (21) and showed a sensitivity of 80% and specificity of 75% [Area under the Curve 0,886 (IC95%, 0,826-0,947)] for mild cognitive impairment using a cutoff of <26 points for abnormal performance (21). For the secondary analysis, we explored each domain separately. The MoCA test was originally developed as a brief tool for primary care physicians to identify elderly patients which might suffer cognitive impairment but perform within the normal range of dementia screening tools (20). Still, it has been used in research settings as a screening tool in younger adults with sleep disorders (22), heart failure (23), Parkinson's Disease (24), Vascular Cognitive Impairment (25), and Systemic Lupus Erythematosus (26). Moreover, since the MoCA tests evaluates different domains in cognitive functions, it has also been used to assess specific areas of dysfunction. In recent studies, it has been observed that a neuroanatomical correlation between MoCA scores sub-specific domains and cortical volumes, particularly in hippocampal area (27).

We assessed disability using the World Health Organization Disability Assessment Schedule 2.0 (WHODAS 2). The WHODAS 2 is an instrument designed to provide a cross-cultural standardized method for measuring activity limitations and participation restrictions irrespective of the individual's medical diagnosis (28).

### Ethical Considerations

Study protocol was evaluated and approved by the Institutional Review Board in all Mexican participating institutions. Participation was voluntary and documented through a written informed consent procedure. Participants younger than 18 years were requested their assent and parents or legal tutors authorized their participation. *This study was carried out in accordance with* the ethical standards of the institutional research committee and with the 1964 Helsinki Declaration and its later amendments or comparable ethical standards.

### Statistical Analysis

We modeled the longitudinal behavior of education-adjusted MoCA scores across study visits, and compared these results between disease groups (Zika, dengue, and AIUO), using a regression model and generalized estimable equations (GEE) to account for intrasubject correlation using the geepack package (29) in R (30). We modeled the MoCA score as function of the indicators for disease group, study visits, education status, sex and age; allowing for interactions between visit and disease group, and then dropped non-significant interactions from the final model. We used contrasts to determine whether there were differences in MoCA scores at each study visit across disease groups. In a secondary analysis, the MoCA score was dichotomized into normal or abnormal (≤ 26 points), and fitted a similar model using logistic regression with GEE. We used the same approach of the continuous MoCA to test time and group difference for five MoCA domain scores that make up the total MoCA score. The longitudinal model assumes data is missing at random. We performed analyses to assess missingness, and a sensitivity analysis using the last rank carried forward (LRCF) method (31), to determine the potential impact of missing data in our final results.

## Results

### Characteristics of the Study Population

We analyzed information from 406 patients of which 73 had confirmed dengue, 37 Zika, and 296 had an acute illnesses of unidentified origin (AIUO). The demographic characteristics of these patients are summarized in Table 1. Overall, patients with dengue were younger and predominantly placed in the lower educations groups (70% with twelve years of school or lower). A brief description of the main clinical characteristics is shown in Table 2. Overall, patients with dengue virus infection and AIUO more frequently presented fever, arthralgia, headache, malaise, behavior alterations, and disability at baseline than patients with ZIKV; while rash was more frequent in ZIKV and dengue virus infection patients compared with those with AIUO.

**Table 1:** Demographic characteristics of participants at baseline visit by disease group (*N* = 406).

**Characteristic**	**Patients, No. (%)**			***p*-value**
	**Zika**	**Dengue**	**AIUO**	
	***n* = 37 (9.11)**	***n* = 73 (17.98)**	***n* = 296 (72.91)**	
Age, median (IQR)	33 (13)	27 (20)	32 (22.25)	0.003
Female	23 (62.16)	36 (49.32)	187 (63.18)	0.095
Male	14 (37.84)	37 (50.68)	109 (36.82)	
Education Level				0.029
Did not go school	2 (5.41)	2 (2.74)	11 (3.72)	
Grades 1–6 (Primary)	4 (10.81)	17 (23.39)	34 (11.49)	
Grades 7–9	4 (10.81)	14 (19.18)	52 (17.57)	
Grades 10–12	9 (24.32)	18 (24.66)	49 (16.55)	
University	16 (43.24)	14 (19.18)	117 (39.53)	
Postgraduate	2 (5.41)	8 (10.96)	33 (11.15)	

*Comparisons were made using Kruskall-Wallis for continuous variables and Fisher's exact Test for categorical variables*.

**Table 2:** Distribution and characteristics of baseline, self-reported signs, and symptoms of patients 12 years and older seeking care within 7 days of onset due to acute episodes of fever and/or rash (*N* = 406).

**Symptoms**	**Confirmed zika infection(*n* = 37)**	**Confirmed dengue infection (*n* = 73)**	**Acute illnesses of unidentified origin (*n* = 296)**	***p*-value^b^**
Fever (>37.2° C)	26 (70.3%)	69 (94.5%)	248 (83.8%)	0.0058
Rash (self-reported)	22 (59.5%)	40 (54.8%)	106 (35.8%)	0.0009
Arthralgia	19 (51.4%)	62 (84.9%)	244 (82.4%)	0.0001
Myalgia	28 (75.7%)	61 (83.6%)	243 (82.1%)	0.551
Conjunctivitis^a^	17 (45.9%)	26 (35.6%)	123 (41.6%)	0.516
Headache	28 (75.7%)	71 (97.3%)	249 (84.1%)	0.0008
Malaise	28 (75.7%)	70 (95.9%)	272 (91.9%)	0.0033
Confusion/Disorientation^c^	3 (8.3%)	7 (11.3%)	72 (25.3%)	0.0058
Behavior Alterations^c^	5 (13.9%)	10 (16.1%)	64 (22.5%)	0.3778
Disability^d^	28.33 (19.165)	40 (24.1675)	33.33 (18.33)	0.0007

a *There was missing data about days of onset of conjunctivitis in two patients with dengue and six patients with undefined fever episodes*.

b *p-values are unadjusted*.

c *Data available only for 383 participants (36 with ZIKV, 62 with DENV, and 285 with AIUO)*.

d *Disability was assessed using the World Health Organization Disability Assessment Schedule 2.0 (WHODAS 2)*.

*Comparisons were made using Kruskall-Wallis for continuous variables and Fisher's exact Test for categorical variables*.

### MoCA Score

**Figure 1:**
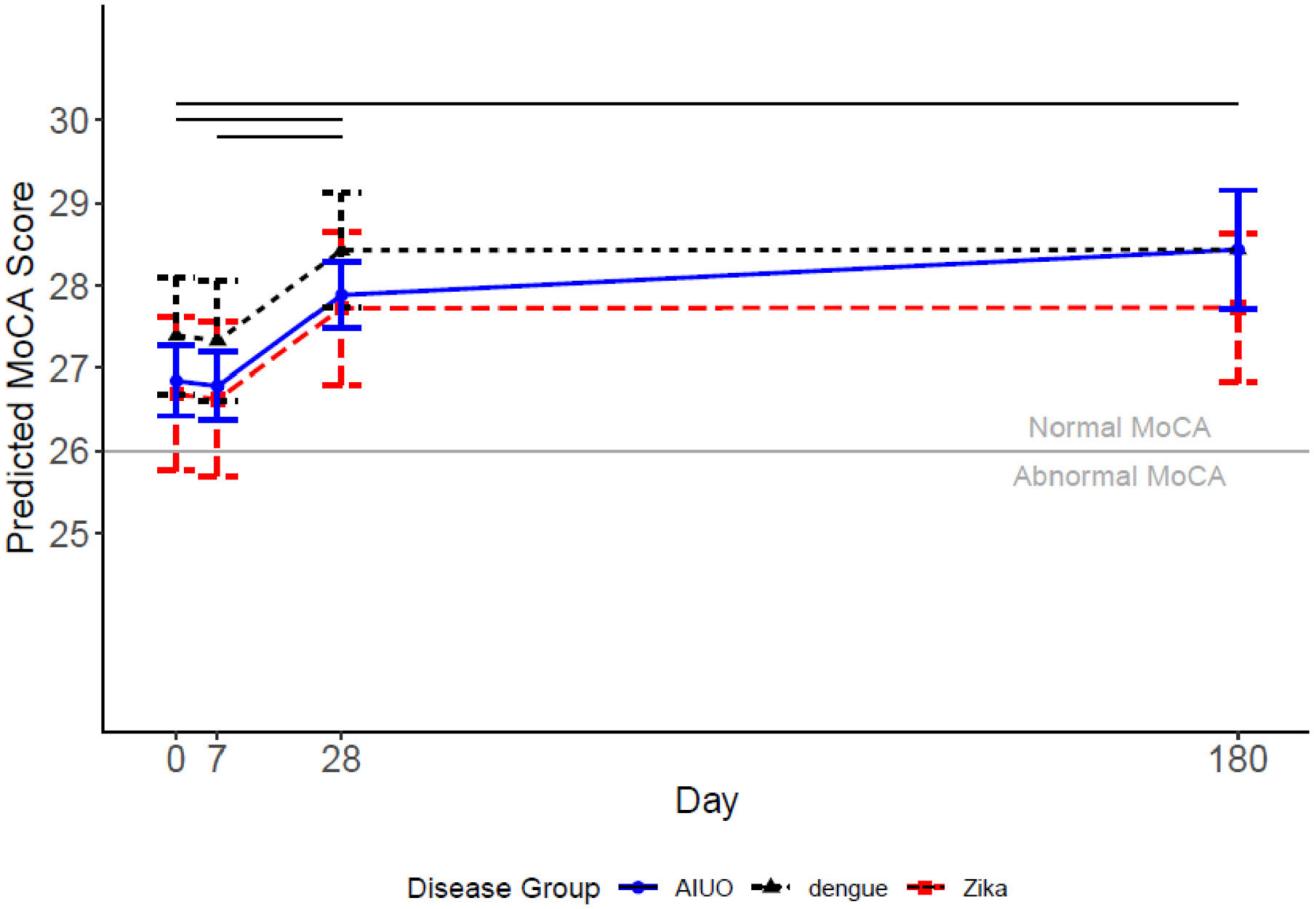
Predicted MoCA scores for each disease group and 95% confidence intervals. Covariates set to 33-year-old female with university education for each disease group. Levels of the adjustment variables were selected to be the most common when categorical and average when continuous. The solid horizonal lines represent significant changes in the level of the score for time comparisons.

When analyzing the MoCA score as a continuous endpoint we observed that all disease groups (Zika, dengue and AIUO) followed similar trajectories. While patients with dengue tended to have higher mean MoCA scores than patients with Zika and AIUO, these differences were not statistically significant neither in general nor at any timepoint (see Figure 1). This slightly higher MoCA score in patients with dengue could be attributed to better performance in the Memory subdomain for this group (see below). Overall, there were no significant changes in the mean MoCA score from baseline to visit at day 7 (−0.06, 95% CI: −0.35, 0.22 *p* = 0.68). There was a significant improvement in the mean MoCA scores between day 7 and 28 (1.10 0.83 1.36, *p* < 0.001), but no further improvement was observed between day 28 and 180 (0.01 −0.31 0.32, *p* = 0.96). Patients with AIUO as a group, but not those with Zika or dengue, had a non-statistically significant increase in the mean MoCA scores from day 28 to day 180, as illustrated in Figure 1. These findings are consistent with the analysis of the MoCA scores used as a binary variable, where we estimated the probabilities of having an abnormal MoCA score (score <26) during follow up. In all groups, the probability of an abnormal MoCA score decreased at days 28 and 180 in comparison to measurements at baseline and day 7 (see Supplementary Table 1). Also, both the Zika and AIUO groups had increased odds of an abnormal MoCA scores in comparison with the dengue group (see Table 3).

**Table 3:** Comparisons of the probabilities of having and abnormal MoCA Score and *p*-values for the group comparisons using the binary MoCA score (abnormal < = 26 points).

	**Estimated Odds (95% CI)**	***p*-value**
Zika–Dengue	2.58 (1.14–5.81)	0.02
Zika–AIUO	1.20 (0.63–2.27)	0.58
Dengue-AIUO	0.46 (0.27–0.81)	0.01

### MoCA Subdomains

**Figure 2:**
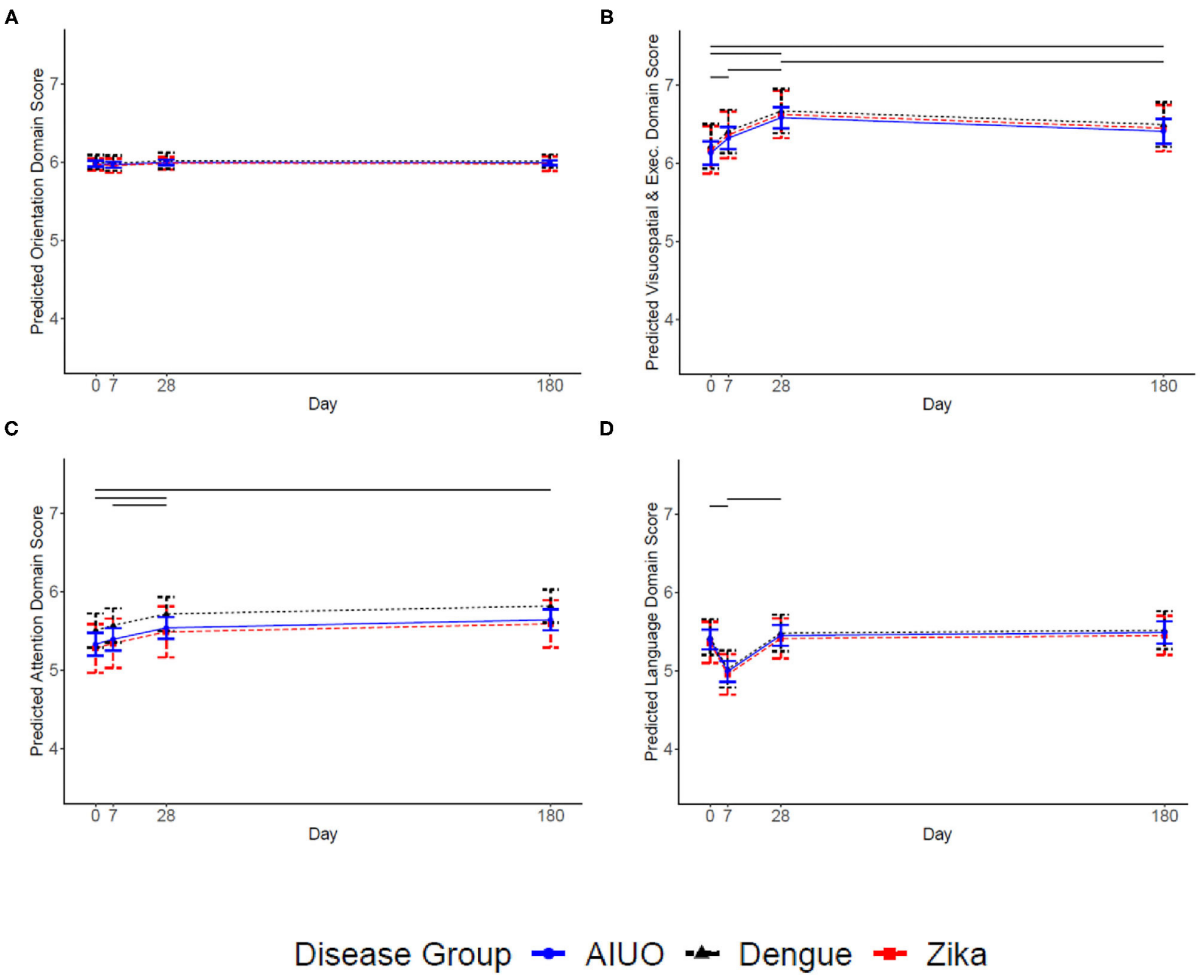
Predicted MoCA Domain scores for each disease group and 95% confidence intervals. Covariates set to 33 year-old female with university education for each disease group. The solid horizonal lines represent significant changes in the level of the domain score between time points. **(A)** Presents Orientation, **(B)** Visuospatial and Executive, **(C)** Attention, and **(D)** Language. The solid horizonal lines represent significant changes (*p* ≤ 0.05) in the score between time-points.

**Figure 3:**
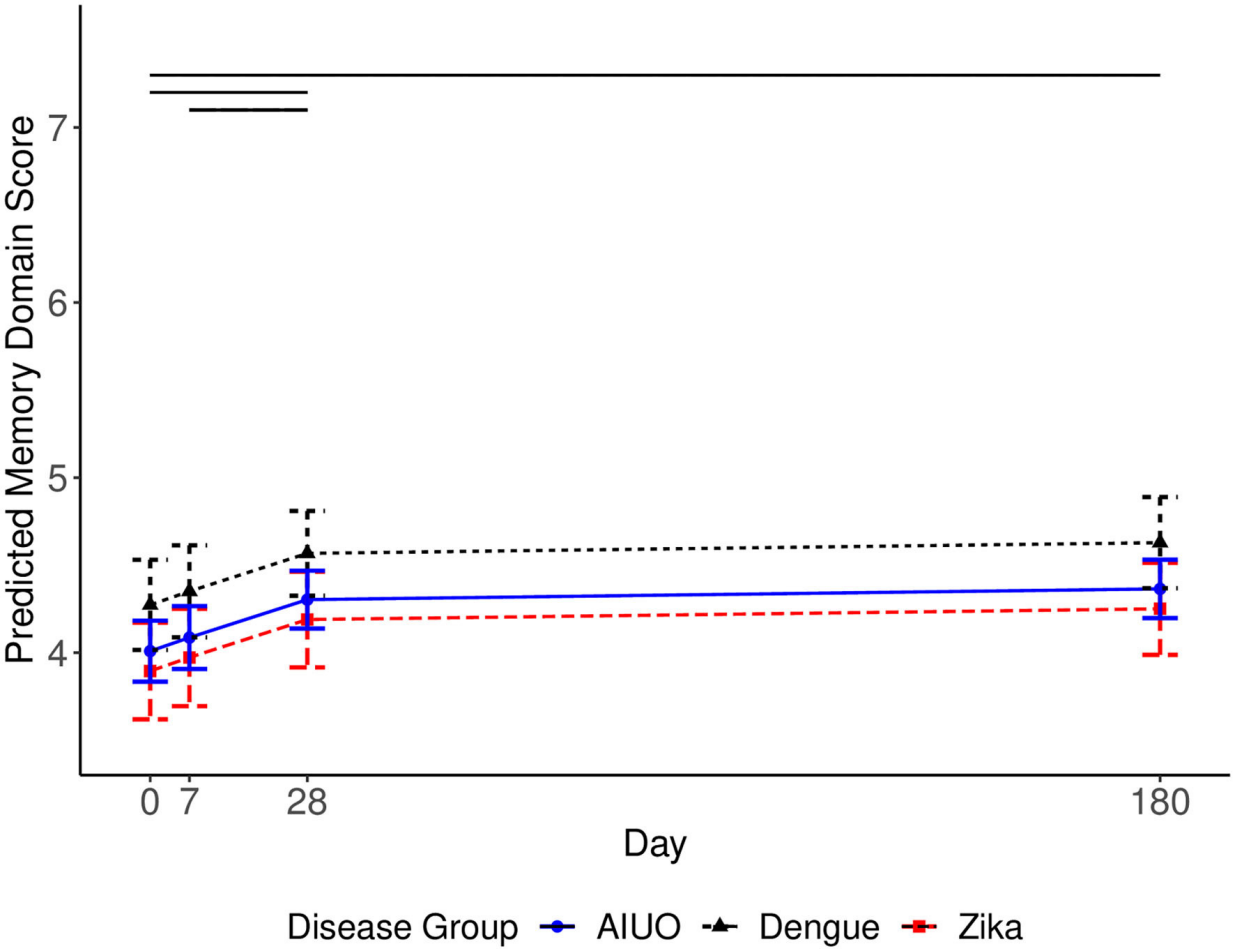
Predicted MoCA Memory Domain scores for each disease group and 95% confidence intervals. Covariates set to 33-year-old female with university education for each disease group. The solid horizonal lines represent significant changes in the level of the domain score between time points. The solid horizonal lines represent significant changes (*p* ≤ 0.05) in the score between time-points.

When we analyzed and compared separately the MoCA test subdomains, we observed no overall differences in orientation, attention, language, and visuospatial and executive functions across disease groups (Figure 2). During follow-up, the orientation subdomain score did not change across time (Figure 2A). The Visuospatial and Executive domain score increased at each follow up time point up to day 28: baseline to day 7 [0.192, (0.073, 0.310), *p* = 0.001], day 7 to day 28 [0.261, (0.116, 0.360), *p* < 0.001], and slightly decreased afterwards: day 28 to 180 [0.277, (0.142, 0.411) *p* ≤ 0.001], but not to the level present at baseline [0.279, (0.136, 0.421, *p* < 0.001)] (Figure 2B). The attention subdomain score did not significantly change between baseline and day 7 [0.06, (−0.05, 0.18) *P* = 0.26], increased from day 7 to day 28 [0.145, (0.04, 0.250), *p* = 0.007], and remained stable from day 28 to day 180 [0.10 (−0.01, 0.22); *P* = 0.08] (Figure 2C). There was an earlier decrease in the language domain score (baseline to day 7 [−0.409, (−0.514, −0.304), *p* < 0.001]) and subsequent recuperation from day 7 to 28 [0.460, (0.355, 0.565), *p* < 0.001], and plateaued from day 28 to 180 [0.037, (−0.079, 0.153), *p* = 0.533] ending at a similar mean score at the end of follow-up than at enrollment (Figure 2D). In contrast, patients with Zika had a lower memory score than patients with dengue (−0.378, 95% CI = −0.678 to −0.078; *p* = 0.014); and patients with dengue had a higher memory score than patients with AIUO [0.264, (0.059, 0.469); *p* = 0.012] (Supplementary Table 2). We observed no differences between the Zika and AIUO groups [−0.114, (−0.369, 0.144); *p* = 0.383]. The difference in the memory subdomain accounted for most of the magnitude of the difference between dengue and Zika or AIUO in the overall MoCA score (Figure 3).

Finally, we observed low memory subdomain mean scores at baseline with no significant changes during the first 7 days. The memory subdomain score increased from day 7 to day 28 [0.217, (0.07, 0.364), *p* = 0.004], and maintained from day 28 to day 180 (Figure 3).

We analyzed missing data patterns among disease groups, which is described in detail in Supplementary Figure 1. Although there is significantly more missing data in the AIUO and dengue groups, there does not appear to be a systematic missingness pattern with respect to baseline MoCA score and disease group. So, it is reasonable to accept the assumption that this information is missing at random.

## Discussion

In this study we present a longitudinal assessment of neurocognitive function, using the MoCA score and its subdomains, of a group of patients with Zika and compared them with patients with dengue and acute illnesses of unidentified origin (AIUO) in the region of Tapachula in Chiapas, Mexico during 2016-2018. We documented a transient deficit in cognitive functions in adults infected with Zika, dengue or AIUO during the first days and up to 28 days after infection with recovery at 6 months of follow-up. While all groups experienced this pattern, patients with Zika and AIUO tended to have a lower overall MoCA score during acute infection and early after that than patients with dengue, although these differences did not reach statistical significance. The probability of having an abnormal MoCA score (<26 points) was significantly higher in patients with Zika and AIUO than in those with dengue. The low memory performance in patients with Zika and AIUO accounts for most of the differences in the overall MoCA score when compared with patients with dengue. The sixth month measurements were higher in patients with AIUO and dengue than in patients with Zika, after adjusting for sex, age and educational status, but again these differences were not statistically significant.

Flaviviruses can cause a wide variety of neurological manifestations (1) including alterations in sensitivity, cognitive impairment, seizures and personality disorders such as mania, depression, emotional lability, anxiety, psychosis and agoraphobia in dengue (32) and encephalitis, myelitis, confusion and paresthesia in patients with Zika (5). We observed transient alterations in attention, visuospatial and memory functions as assessed by the MoCA test in all groups of patients, suggesting a possible common mechanism of neural damage instead of specific to each disease. Considering the known mechanisms of neural damage by flaviviruses, our findings could be explained by direct viral replication in brain tissue with subsequent neuronal destruction, immune complex formation or both (32, 33). It also has been demonstrated that neural damage may result from the preexistence of cross-reactive antibodies against flavivirus during ZIKV infection (34–36). Interestingly, patients with Zika and AIUO had a lower memory score than patients with dengue during acute infection and this difference persisted over the 6 months of observation. Animal models showing Zika virus infection and viral replication in the frontal cortex and hippocampus associated with synapse damage leading to memory alterations (37), as well as the presence of ZIKV in CSF in patients with overt encephalopathy (38) are consistent with direct damage by ZIKV to adult neurons (39).

Our study has several drawbacks which might limit the validity of our results. First, it comprises a relatively small group of people with Zika and dengue with most participants in the AIUO group. The latter might include an heterogenous group of infections, which limits our ability to have a comparison group clearly differentiated from Zika, dengue and other flaviviruses. We reasonably excluded the possibility of a potential confounding effect of differentiated patterns of missing data due to loss of follow-up associated with cognitive dysfunction or disease group, and adjusted for the potential confounding effects of sex, age and educational status in our model.

While the MoCA screening test is a convenient, widely used, validated instrument to assess cognitive functions, it was developed to identify mild cognitive impairment in elderly patients performing within the normal range of dementia screening tools. Thus, it might still be poorly sensitive to milder or subclinical cognitive dysfunction in younger people, for whom tools to properly assess cognitive functioning might have been needed. The advantage of the MoCA score is that despite its limitations, its widespread use in research settings facilitates comparisons across studies. Finally, the lack of cerebral neuroimaging studies and cerebrospinal fluid testing, make it difficult to propose a mechanism for the observed changes in memory, visuospatial and attention cognitive domains in all groups. Even so, our study is one of the first to explore the impact of Zika on cognitive functions. Our results show a decrease in cognitive function during acute illness, it would be interesting to follow up on this finding with a larger sample size and more refined tools to identify cognitive impairment.

In conclusion, while Zika is a neurotropic virus that can produce a constellation of severe neurological manifestations, few studies have explored its short- and long-term impact in cognitive function in adult humans. We observed a transient impact on cognitive functions in patients with Zika, dengue and with other undefined acute illnesses, particularly in memory, visuospatial and attention domains. Patients with Zika tended to have lower MoCA scores at 6 months of follow-up than patients with AIUO and dengue but this finding has uncertain clinical significance. Our study provides no clinical evidence to support the hypothesis that Zika or dengue might cause neurocognitive alterations persisting longer than the period of acute infection. While effects on memory or perhaps other cognitive functions over the long term are possible, larger studies using more refined tools for neurocognitive functioning assessment are needed to identify these.

## Data Availability Statement

The raw data supporting the conclusions of this article will be made available by the authors, without undue reservation.

## Ethics Statement

The studies involving human participants were reviewed and approved by Comité de Ética en Investigación (Comittee on Ethics in Research) and the Comité de Investigación (Research Comittee) at the Instituto Nacional de Ciencias Médicas y Nutrición Salvador Zubirán (INF-2636-18-19-1. Written informed consent to participate in this study was provided by the participants' legal guardian/next of kin.

## Author Contributions

PB-Z performed the literature search, was part of the study design team, collected data, wrote sections of the paper, and contributed to data analysis an interpretation. AO-V produced the figures, performed the data analysis and interpretation, and edited the paper. AM-A participated in data analysis and interpretation, wrote sections of the paper and edition. PG-D-B performed the literature search, contributed to data interpretation and the writing and editing of the manuscript. SA-N provided contributed to data analysis and interpretation, wrote sections of the paper and edition. JS-D performed the literature search, wrote sections of the paper, and provided data interpretation. SH wrote sections of the paper, was part of the study design team, and performed the data analysis and interpretation. RV and JR-C participated in data interpretation and edited the paper. HR, JN, SC, and ER was part of the study design team and collected data. PR was part of the study design team and provided data interpretation. JB was part of the study design team and provided supervision. JP provided data interpretation and edited the paper. GR-P and CL was part of the design team, obtained funding and provided supervision. All co-authors critically reviewed the manuscript and approved the final version.

## Conflict of Interest

The authors declare that the research was conducted in the absence of any commercial or financial relationships that could be construed as a potential conflict of interest.
